# Ischemic preconditioning *vs* adenosine *vs* prostaglandin E1 for protection against liver ischemia/reperfusion injury

**DOI:** 10.1590/1414-431X20176185

**Published:** 2017-07-17

**Authors:** M. Radojkovic, M. Stojanovic, G. Stanojevic, D. Radojkovic, J. Gligorijevic, I. Ilic, N. Stojanovic

**Affiliations:** 1 Surgery Department, School of Medicine, University of Nis, Nis, Serbia; 2 Internal Medicine Department, School of Medicine, University of Nis, Nis, Serbia; 3 Pathology Department, School of Medicine, University of Nis, Nis, Serbia; 4 School of Medicine, University of Nis, Nis, Serbia

**Keywords:** Liver ischemia/reperfusion injury, Ischemic preconditioning, Adenosine, Prostaglandin E1, Protection

## Abstract

Ischemia/reperfusion injury is still a major cause of morbidity and mortality during liver surgery and transplantation. A variety of surgical and pharmacological therapeutic strategies have been investigated to minimize the effects of ischemia/reperfusion. The aim of our study was to analyze and compare preventive influences of ischemic preconditioning, adenosine and prostaglandin E1 in the experimental model of hepatic ischemia/reperfusion injury. Adult chinchilla rabbits were divided into four groups: 10 rabbits subjected to liver ischemic preconditioning (3-min period of inflow occlusion followed by a 5-min period of reperfusion) followed by 45 min of Pringle maneuver; 10 rabbits subjected to pre-treatment with intraportal injection of adenosine followed by 45 min of Pringle maneuver; 10 rabbits subjected to pre-treatment with intraportal injection of prostaglandin E1 followed by 45 min of Pringle maneuver; and control group of 10 rabbits subjected to 45 min of inflow liver ischemia without any preconditioning. On the second postoperative day, blood samples were obtained and biochemical parameters of liver function were measured and compared. Liver tissue samples were also obtained and histopathological changes were compared. Based on biochemical and histopathological parameters, it was demonstrated that ischemic preconditioning provided the best protection against hepatic ischemia/reperfusion injury. This was probably due to a wider range of mechanisms of action of this method oriented to reduce oxidative stress and inflammation, and restore liver microcirculation and hepatocyte energy compared to the examined pharmacological strategies.

## Introduction

Ischemia/reperfusion injury (IRI) is still a major cause of morbidity and mortality during liver surgery and transplantation leading to a high incidence of complications, such as graft dysfunction and rejection, respectively. In this biphasic process, initial hepatocellular damage due to hypoxia is further aggravated upon restoration of oxygen supply as a result of complex and diverse interactions between hepatocytes, liver sinusoidal endothelial cells, Kupffer cells, hepatic stellate cells with various inflammatory cells and mediators, and reactive oxygen species leading to subsequent biochemical disturbances in intracellular homeostasis (1,2). A variety of surgical and pharmacological therapeutic strategies have been investigated to minimize the effects of IRI and improve postresection/transplant outcome. The protective effect of ischemic preconditioning (IP), introduced by Murry et al. (3) in the myocardial IRI model, was subsequently confirmed in the liver as well (4). Among numerous agents, it was reported that exogenous adenosine (AD) attenuates the hepatic IRI by preventing the decrease in nitric oxide (NO) production (5). Also, a protective mechanism of prostaglandin E1 (PGE1) against liver IRI by reducing leucocyte-endothelial cell adhesion was demonstrated *in vivo* (6). The aim of our study was to analyze and compare preventive influences of IP, adenosine and PGE1 in the experimental model of hepatic IRI.

## Material and Methods

### Experimental animals

The experiment was done using healthy adult chinchilla rabbits, of both genders, weighing from 1.2 to 2.5 kg (average 1.77) and aged 1.5–3 months (average 2.5). Animals were divided into four groups: IP group with 10 rabbits subjected to anesthesia and liver IP (3-min period of inflow occlusion followed by a 5-min period of reperfusion) followed by 45 min of Pringle maneuver; AD group with 10 rabbits subjected to anesthesia, pre-treatment with intraportal injection of 1 mg/kg exogenous adenosine (Serva Electrophoresis GmbH, Germany) dissolved in bicarbonate-buffered saline (pH 7.4) and followed by 45 min of Pringle maneuver; PGE1 group with 10 rabbits subjected to anesthesia, pre-treatment with intraportal injection of 0.5 µg/kg PGE1 (Alprostadil Alfadex, Prostavasin¯ 20 µg, Schwarz Pharma AG, Germany) and followed by 45 min of Pringle maneuver; and control group (CG) of 10 rabbits subjected to anesthesia and 45 min of inflow liver ischemia without any preconditioning. None of the animals received medication preoperatively.

### Surgical procedures

Rabbits were anesthetized with an intramuscular injection of 15 mg/kg tiletamine hydrochloride and zolazepam hydrochloride combination (Zoletil¯ 50, Virbac S.A., France). A midline incision was performed and the surgical or pharmacological preconditioning were carried out. The hepatic pedicle was clamped using a De Bakey Bulldog Clamp (Sklar Surgical Instruments, USA). The procedures and postoperative course were uneventful.

### Biochemical assay

Blood samples were taken on the second postoperative day by puncture of the left myocardial ventricle through the midline thoracoabdominal incision. Serum total protein (TP) level and activities of alanine transaminase (ALT), aspartate transaminase (AST), lactate dehydrogenase (LDH), alkaline phosphatase (ALP) and gamma-glutamyltransferase (GGT) were measured and detected values were compared.

### Histopathological (HP) examination

On the same occasion, liver tissue samples were also taken, fixed with 10% formaldehyde, stained using hematoxylin and eosin (HE), periodic acid-Schiff and reticulin stain protocols and analyzed with light microscopy. Observed HP changes caused by liver IRI were compared.

### Statistical analysis

Statistical analysis was performed using SPSS version 15.0 software (IBM, USA). Continuous variables are reported as means±SD and medians. The distributions of the continuous variables were assessed for normality by Shapiro-Wilk test. The differences of continuous variables between the two independent groups were compared using Student's *t*-test and the Mann-Whitney U-test, as appropriate, and between more than two groups using one-way ANOVA or Kruskal-Wallis test, depending on data distribution. In the case of one-way ANOVA, multiple comparisons were performed, using appropriate post-hoc tests. Statistical significance was defined as P<0.05.

All the experimental procedures were carried out according to the basic principles of laboratory animal care and Serbian Law on animal welfare and after approval from Ethics Committee at School of Medicine, University of Nis, Serbia.

## Results and Discussion

The results of the biochemical assay are presented in Table 1. The serum values of all analyzed biochemical parameters were statistically significant, depending on ischemic and pharmacologic liver preconditioning (P<0.001 for TP, ALT, AST, and LDH; P<0.05 for ALP), with the exception of GGT. Compared to CG rabbits subjected to IRI without protection, serum TP levels were significantly higher in IP and AD groups (P<0.001) and lower in the PGE1 group (P<0.05). TP levels in PGE1 group were also significantly lower in comparison to both IP and AD groups (P<0.001). Since serum TP is an indicator of liver synthetic function its elevated values suggest strong protective effects of IP and AD and, accordingly, lack of protection against IRI by PGE1. This is not consistent with previous reports (7) and it may be explained with the very rapid metabolism and extraction of PGE1 by the hepatocytes and lungs (1.24 min) (8). Therefore, the continuous intravenous administration of this agent is mandatory (9). It may also have contributed to the PGE1 anti-inflammatory action and consequentially decreased production of globulins, especially gamma globulins in lymphocytes and plasma cells, leading to lower TP levels (10).

**Table 1.** Serum biochemical parameters of animals treated with ischemic preconditioning (IP), adenosine and prostaglandin E1 (PGE1) in the experimental model of hepatic ischemia/reperfusion injury.

	Adenosine	PGE1	IP	Control
TP***	73.33±4.73*** b d	53.58±2.74	75.22±3.27*** b d	55.84±1.99* b
	(72.70)	(52.65)	(76.25)	(55.75)
AST***	49.10±3.48	78.40±3.63*** a c	47.20±2.53	102.40±7.49*** a b c
	(49.50)	(79.50)	(47.50)	(100.50)
ALT***	79.50±2.92	120.70±5.87*** a c	78.70±5.01	201.60±13.24*** a b c
	(80.50)	(119.50)	(79.50)	(200.50)
LDH***	149.40±7.79	185.50±6.11*** a c	159.40±5.19* a	234.70±19.66*** a b c
	(148.50)	(185.50)	(159.50)	(235.50)
ALP*	323.55±91.47* b c	261.75±167.44	232.51±68.63	301.05±59.32* b c
	(310.60)	(195.60)	(221.50)	(281.80)
GGT	12.38±4.63	9.23±3.75	8.88±2.44	11.93±3.70
	(13.00)	(8.55)	(8.90)	(12.45)

Data are reported as means±SD (medians). The control group did not receive preconditioning. TP: serum total protein; AST: aspartate transaminase; ALT: alanine transaminase; LDH: lactate dehydrogenase; ALP: alkaline phosphatase; GGT: gamma-glutamyltransferase.

* P<0.05;

** P<0.01;

*** P<0.001,

a *vs* adenosine;

b *vs* PGE1;

c *vs* IP,

d *vs* Control. Comparisons between all four groups: one-way ANOVA or Kruskal-Wallis test. Comparisons between two independent groups: in cases of ANOVA, multiple comparisons with appropriate *post hoc* tests; in case of Kruskal-Wallis test, Student's *t*-test or the Mann-Whitney U-test, depending on data distribution.

An identical pattern of alterations was demonstrated for ALT, AST, and LDH. Serum activities of these biochemical parameters were markedly higher in CG animals compared to the other three groups (IP, AD and PGE1; P<0.001). Also, mean values of all three enzymes were significantly higher in rabbits treated with PGE1 in comparison with animals treated with IP and AD (P<0.001). Furthermore, LDH serum activity was significantly higher in IP than AD group (P<0.05). These enzymes are considered the most sensitive markers of hepatocellular damage and necrosis accompanied with increased permeability of both cell and intracellular membranes. Considering that CG rabbits were not subjected to any preconditioning, elevated serum ALT, AST and LDH values were expected. Therefore, significantly higher activities of these enzymes in PGE1 rabbits compared to IP and AD animals indicated less effective amelioration of liver IRI with PGE1 preconditioning in comparison with IP and AD. While ALT is localized solely in the cellular cytoplasm, AST and LDH are both cytosolic and mitochondrial enzymes (11,12). Hence, considering the significantly higher LDH serum activity in the IP group compared to the AD group, this would suggest a greater hepatocellular damage, including the release of both cytosolic and mitochondrial LDH by mitochondria in IP animals, i.e. weaker protection provided by IP.

### Protection of microcirculation and energy metabolism

Protective effects of IP and AD are the result of their action on preservation of liver microcirculation and hepatocyte energetic metabolism, and on reduction of inflammatory response and oxidative stress. IP induces slower adenine nucleotides degradation and adenosine triphosphate (ATP) consumption, thus decreasing cellular energy demand during ischemia (13). A possible mechanism of this energy-sparing action may include the inhibition of glycolysis by adenosine monophosphate (AMP)-activated protein kinase, which is increased during IP due to ATP degradation and ATP-induced inhibition of pyruvate kinase, an important regulatory enzyme in glycolysis (14). Also, IP induces the so-called “metabolic membrane arrest”, i.e. significant suppression of non-essential cellular activity (15), and increases efficiency of phosphotransfer networks (energy relays that link sources of energy production with sites of their utilization) enabling effective movement of ATP across mitochondria through enzyme kinetics and ion transfer (16) and thus improving energetic metabolism and ischemia tolerance. Along with the energy-sparing action, IP exerts a direct effect on maintaining intracellular ion homeostasis and acid-base balance. It also prevents cell swelling and rupture due to Na^+^/K^+^ ATPase pump failure and Na^+^ and Ca^2+^ influx from the extracellular space during IRI induced by ATP depletion (17). Moreover, IP reduces calcium accumulation in the matrix by keeping the mitochondrial K_ATP_ channels open during ischemia and maintaining ionic equilibrium across mitochondrial membranes, which contributes to cellular integrity (18).

### Prevention of oxidative stress and “no-reflow” phenomenon

In hypoxic tissue, xanthine dehydrogenase (XD) converts to xanthine oxidase (XO) in endothelial cells. During reperfusion, large amounts of free oxygen radicals is produced as a result of xanthine degradation by XO. Reactive oxygen species (ROS) significantly contribute to reperfusion injury by activation of leucocytes and lipid peroxidation of membranes. It is reported that IP reduces conversion of XD to XO (19), activation of Kupfer cells (great producers of ROS) and leucocyte-endothelial interaction, and preserves mitochondrial redox potential, thus attenuating the oxidative stress during reperfusion (20).

After reperfusion, the “no-reflow” phenomenon occurs leading to endothelial cell swelling, activation of adhesion molecules, platelets and neutrophils, and reduction of NO due to the lack of perfusion of up to one third of the vascular bed. IP decreases leucocyte adhesion via downregulation of inter-cellular adhesion molecule 1 production (21). It also reduces vasospasm, preserves endothelial vasoregulatory function and prevents microcirculatory deficit by enhancement of production and metabolism of important vasodilating factors, such as arachidonic acid, eicosanoids and NO. Among numerous mechanisms, IP increases the production of endogenous AD, which induces the production of vasodilator NO by activation of A1 and A2 adenosine receptors, leading to the inhibition of hepatic sinusoidal vasoconstriction (20). The effects of IP are mediated by AMP-activated protein kinase (22). NO inhibits the effects of the strong vasoconstrictor peptide endothelin. Furthermore, heme oxygenase (HO)-1, an enzyme involved in heme degradation catalyzing the formation of carbon monoxide (CO), biliverdin and Fe^2+^ ions, is induced under IRI and acts cytoprotective via vasodilating, anti-apoptoic and anti-necrotic effects of CO and biliverdin (23).

In addition to the vasoregulatory action, NO is an effector molecule involved in regulation of immune response, inhibiting proinflammatory cytokines that induce the inflammatory reaction during IRI such as tumor necrosis factor (TNF)-α, interleukin (IL)-1α, IL-1β and IL-12 (24). Its anti-inflammatory role also includes numerous effects on immune cells: inhibition of T helper (Th)1 cell and enhancement of Th2 cell proliferation, reduction of leucocyte recruitment, and adhesion and assistance to T regulatory cells. Similarly, the activation of A1 and A2a adenosine receptors after IRI produces cytoprotection via anti-inflammatory, anti-apoptotic and anti-necrotic effects. AD promotes the efflux of Ca^2+^ accumulated during ischemia out of both hepatocytes and hepatic microcirculation smooth muscle cells, leading to cell membrane stabilization and microcirculatory vasorelaxation, and preventing Ca^2+^ overload and cell death (25). It acts via A1 receptor, reducing neutrophil infiltration and their adherence to microvascular endothelium (26). In addition to that, the protective action of AD is probably also mediated by NO, a potent agent inhibiting the activation of pro-inflammatory transcription factors and subsequent expression of pro-inflammatory cytokines, chemokines and adhesion molecules.

The effects of PGE1 are predominantly anti-inflammatory and slightly differ from IP and AD. It inhibits tumor necrosis factor alpha release from Kupffer cells, adhesion molecule expression and neutrophil adherence to endothelial cells, and has antiaggregation and fibrinolytic activity (10,27,28). It would seem that the mechanisms of action of IP and AD oriented to liver microcirculation and hepatocyte energy provide better protection against liver IRI. Also, IP and exogenous AD might share the same metabolic pathway of cytoprotection. During ischemia, ATP degradation increases, ultimately leading to intracellular AD accumulation. Intracellular AD further converts to inosine and both AD and inosine come out of cells. Extracellularly accumulated AD acts protectively, and during reperfusion, it is used for ATP resynthesis and cell energy restoration. IP reduces IRI by increasing the production of endogenous AD in the liver. Sufficient concentrations of both endogenous and exogenously administered AD provide effective cytoprotection.

The alterations of ALP serum activities in experimental rabbits were significantly dependent on the preconditioning method (P<0.05), while GGT values were not. In AD group, the activities of both enzymes were higher than CG, while in IP and PGE1 groups they were lower than CG. The lowest values of both enzymes were measured in IP group and were significantly lower in comparison with CG (P<0.05 for ALP and no significance for GGT). Both ALP and GGT are present on the surface of bile duct epithelia and in hepatocytes, and are generally considered indicators of cholestasis. Therefore, the lowest values of both enzymes detected in IP animals may suggest good protection of IP against cholestatic damage and the lack of this effect in AD group. It was suggested that activation of A2a adenosine receptors mediates the hepatoprotective effects of IP (5). However, Kim et al. reported that activation of A1 receptors by exogenous AD failed to attenuate liver IRI in an experimental model (29), which may be the reason for the poorer protective action of AD. Moreover, the 45-min extrahepatic biliary obstruction may not be sufficient to induce biochemically relevant cholestasis. Additionally, choleresis and bile flow restoration and recovery from cholestasis occur much faster than improvement of hepatic microcirculation and recovery of IRI, which may diminish the informative relevance of ALP and GGT as markers of IRI. This is consistent with previous reports on insignificant ALP and GGT changes after prolonged inflow liver occlusion (30). Nevertheless, further research with longer follow-up investigating the long-term effects of these protective strategies against IRI are needed.

### Histopathological alterations

**Figure 1.**
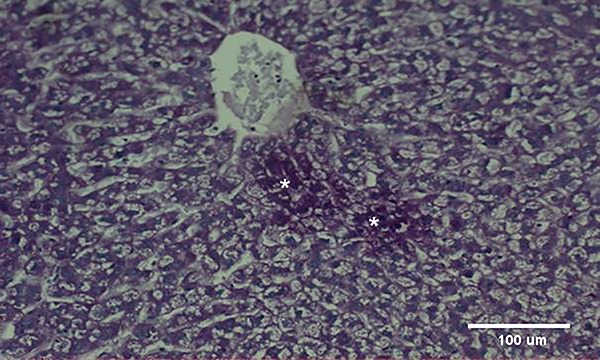
Spot necrosis (marked with asterisks) and vacuolar degeneration in liver tissue of control group rabbits (periodic acid-Schiff staining).

**Figure 2.**
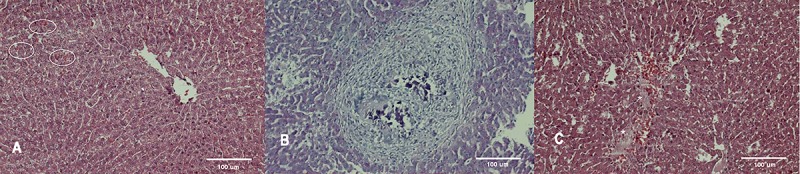
*A*, Microhemorrhage (circled in white) (hematoxylin and eosin). *B*, Myelin figures in the central part of the image indicating necrosis (periodic acid-Schiff staining). *C*, Micronecrosis (hematoxylin and eosin) in liver tissue of prostaglandin E1-treated rabbits (marked with asterisks).

**Figure 3.**
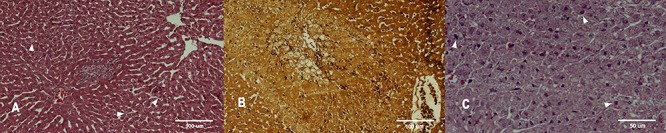
*A*, Focal vacuolar degeneration in ischemic preconditioning group (marked with arrowheads) (hematoxylin and eosin). *B*, Necrobiosis in the central part of the image in adenosine group (reticulin). *C*, Regeneration zone: hepatocytes with hyperchromic nuclei in adenosine group rabbit (marked with arrowheads) (hematoxylin and eosin).

The observed HP changes were consistent with the results of the biochemical assay. As expected, the most intense HP changes were present in liver tissues of CG rabbits and included different types of degeneration, hepatocyte coagulative necrosis, fresh bleeding, venous stasis and inflammation (Figure 1). The same range of alterations was found in samples of animals pre-treated with PGE1, only they were less pronounced: venous congestion and bleeding were less frequent and inflammatory infiltrates were fewer (Figure 2). Nevertheless, necrosis, although solitary and less intense (micronecrosis) compared to CG group, was also present. However, no hemorrhage and necrosis were found in liver samples of rabbits pre-treated with IP and adenosine, with vacuolar and granular degeneration in the perivenular zones and mild to moderate inflammation being the prevailing alterations. Kupffer cells hyperplasia and focal macrophage infiltration in the portal spaces suggested activated immune response in these rabbits. In addition, sporadic hepatic necrobiosis observed in samples of several AD rabbits was the most severe finding and indicated gradual, less fulminant development of IRI compared with CG and PGE1 animals. Also, enlarged hepatocytes with hyperchromic nuclei were present only in some samples of IP and AD rabbits suggesting regeneration and recovery (Figure 3). Less severe and gradual HP changes associated with the signs of activated immunity and regeneration clearly demonstrated stronger protective action against IRI of IP and adenosine *versus* PGE1.

### Conclusion

In the experimental model with rabbits, IP, PGE1 and AD were shown to have protective effects against liver IRI. However, based on biochemical and HP parameters of damage that were analyzed and compared in our study, it seems that IP provided the best protection against hepatic IRI compared to adenosine and PGE1, considering the lack of preventive effects of adenosine towards cholestatic disturbances. This is probably due to the wider range of mechanisms of actions of IP towards the reduction of oxidative stress and inflammation, and restoration of liver microcirculation and hepatocyte energy compared to the pharmacological strategies.

## References

[1] Montalvo-Jave EE, Escalante-Tattersfield T, Ortega-Salgado JA, Pina E, Geller DA (2008). Factors in the pathophysiology of the liver ischemia-reperfusion injury. J Surg Res.

[2] Gracia-Sancho J, Villarreal G, Zhang Y, Yu JX, Liu Y, Tullius SG (2010). Flow cessation triggers endothelial dysfunction during organ cold storage conditions: strategies for pharmacologic intervention. Transplantation.

[3] Murry CE, Jennings RB, Reimer KA (1986). Preconditioning with ischemia: a delay of lethal cell injury in ischemic myocardium. Circulation.

[4] Hardy KJ, McClure DN, Subwongcharoen S (1996). Ischaemic preconditioning of the liver: a preliminary study. ANZ J Surg.

[5] Peralta C, Hotter G, Closa D, Prats N, Xaus C, Gelpí E (1999). The protective role of adenosine in inducing nitric oxide synthesis in rat liver ischemia preconditioning is mediated by activation of adenosine A_2_ receptors. Hepatology.

[6] Natori S, Fujii Y, Kurosawa H, Nakano A, Shimada H (1997). Prostaglandin E1 protects against ischemia-reperfusion injury of the liver by inhibition of neutrophil adherence to endothelial cells. Transplantation.

[7] Hsieh CC, Hsieh SC, Chiu JH, Wu YL (2014). Protective effects of N-acetylcysteine and a prostaglandin E1 analog, alprostadil, against hepatic ischemia: reperfusion injury in rats. J Tradit Complement Med.

[8] Garrity MJ, Brass EP, Robertson RP (1984). Kinetics of prostaglandin E metabolism in isolated hepatocytes. Biochim Biophys Acta.

[9] Cawello W, Leonhardt A, Schweer H, Seyberth HW, Bonn R, Lomeli AL (1995). Dose proportional pharmacokinetics of alprostadil in healthy volunteers following intravenous infusion. Br J Clin Pharmacol.

[10] Hafez T, Moussa M, Nesim I, Baligh N, Davidson B, Abdul-Hadi A (2007). The effect of intraportal prostaglandin E1 on adhesion molecule expression, inflammatory modulator function and histology in canine hepatic ischemia/reperfusion injury. J Surg Res.

[11] Rej R (1989). Aminotransferases in disease. Clin Lab Med.

[12] Ketchum CH, Robinson CA, Hall LM, Grizzle WE (1988). Lactate dehydrogenase isolated from human liver mitochondria: its purification and partial biochemical characterization. Clin Biochem.

[13] Jennings RB, Sebbag L, Schwartz LM, Crago MS, Reimer KA (2001). Metabolism of preconditioned myocardium: effect of loss and reinstatement of cardioprotection. J Mol Cell Cardiol.

[14] Pasupathy S, Homer-Vanniasinkam S (2005). Ischaemic preconditioning protects against ischaemia/reperfusion injury: emerging concepts. Eur J Vasc Endovasc Surg.

[15] Stenzel-Poore MP, Stevens SL, Xiong Z, Lessov NS, Harrington CA, Mori M (2003). Effect of ischaemic preconditioning on genomic response to cerebral ischaemia: similarity to neuroprotective strategies in hibernation and hypoxia-tolerant states. Lancet.

[16] Pucar D, Dzeja PP, Bast P, Juranic N, Macura S, Terzic A (2001). Cellular energetics in the preconditioned state: protective role for phosphotransfer reactions captured by 18O-assisted 31P NMR. J Biol Chem.

[17] Carini R, de Cesaris MG, Splendore R, Bagnati M, Albano E (2000). Ischemic preconditioning reduces Na(+) accumulation and cell killing in isolated rat hepatocytes exposed to hypoxia. Hepatology.

[18] Wang L, Cherednichenko G, Hernandez L, Halow J, Camacho SA, Figueredo V (2001). Preconditioning limits mitochondrial Ca(2+) during ischemia in rat hearts: role of K(ATP) channels. Am J Physiol Heart Circ Physiol.

[19] Fernandez L, Heredia N, Grande L, Gomez G, Rimola A, Marco A (2002). Preconditioning protects liver and lung damage in rat liver transplantation: role of xanthine/xanthine oxidase. Hepatology.

[20] Glanemann M, Vollmar B, Nussler AK, Schaefer T, Neuhaus P, Menger MD (2003). Ischemic preconditioning protects from hepatic ischemia/reperfusion injury by preservation of microcirculation and mitochondrial redox-state. J Hepatol.

[21] Shinoda M, Shimazu M, Wakabayashi G, Tanabe M, Hoshino K, Kitajima M (2002). Tumor necrosis factor suppression and microcirculatory disturbance amelioration in ischemia/reperfusion injury of rat liver after ischemic preconditioning. J Gastroenterol Hepatol.

[22] Peralta C, Bartrons R, Serafin A, Blázquez C, Guzmán M, Prats N (2001). Adenosine-monophosphate-activated protein kinase mediates the protective effects of ischemic preconditioning on hepatic ischemia-reperfusion injury in the rat. Hepatology.

[23] Kato Y, Shimazu M, Kondo M, Uchida K, Kumamoto Y, Wakabayashi G (2003). Bilirubin rinse: a simple protectant against the rat liver graft injury mimicking heme oxygenase-1 preconditioning. Hepatology.

[24] Husted TL, Blanchard J, Schuster R, Shen H, Lentsch AB (2006). Potential role for IL-23 in hepatic ischemia/reperfusion injury. Inflamm Res.

[25] Guan LY, Fu PY, Li PD, Li ZN, Liu HY, Xin MG (2014). Mechanisms of hepatic ischemia-reperfusion injury and protective effects of nitric oxide. World J Gastrointest Surg.

[26] Cronstein BN, Rosenstein ED, Kramer SB, Weissmann G, Hirschhorn R (1985). Adenosine: A physiologic modulator of superoxide anion generation by human neutrophils. Adenosine acts via an A1 receptor on human neutrophils. J Immunol.

[27] Currin RT, Reinstein LJ, Lichtman SN, Thurman RG, Lemasters JJ (1993). Inhibition of tumor necrosis factor release from cultured rat Kupffer cells by agents that reduce graft failure from storage injury. Transplant Proc.

[28] Himmelreich G, Hundt K, Neuhaus P, Bechstein WO, Roissant R, Reiss H (1993). Evidence that intraoperative prostaglandin E1 infusion reduces impaired platelet aggregation after reperfusion in orthotopic liver transplantation. Transplantation.

[29] Kim J, Kim M, Song JH, Lee HT (2008). Endogenous A1 adenosine receptors protect against hepatic ischemia reperfusion injury in mice. Liver Transpl.

[30] Elias D, Desruennes E, Lasser P (1991). Prolonged intermittent clamping of the portal triad during hepatectomy. Br J Surg.

